# Research on the impact of carbide slag content on the strength and microstructure of solidified sludge during composite excitation

**DOI:** 10.1371/journal.pone.0314809

**Published:** 2024-12-16

**Authors:** Shunmei Gong, Shiquan Wang, Xiangyi Yang, Haibin Wang, Yili Zheng, Songbao Feng

**Affiliations:** 1 National Engineering Research Center of Coal Mine Water Hazard Controlling, School of Resources and Civil Engineering, Suzhou University, Suzhou, China; 2 Shenzhen General Integrated Transportation And Municipal Engineering Design & Research Institute Co., Ltd, Shenzhen, Guangdong, China; 3 China Resources Wufeng (China) Investment Co., Ltd., Shenzhen, Guangdong, China; 4 Shenzhen Jingtairong Environmental Technology Co., Ltd., Shenzhen, Guangdong, China; National Chung Cheng University, Taiwan & Australian Center for Sustainable Development Research and Innovation (ACSDRI), AUSTRALIA

## Abstract

A composite material was developed using carbide slag, water glass, slag, and micron silicon to facilitate the use of industrial waste resources. The mechanical properties of dredge sludge (DS) were analyzed, considering different proportions of cement, organic debris, and carbide slag. The composition and microstructure of the hydration products were analyzed using the X-ray diffractometer (XRD), scanning electron microscopy (SEM), and thermogravimetric (TG) analysis. The results indicate that with a precursor content of 20%, a water glass content of 3%, and an increase in carbide slag content from 4% to 12%, the strength of the sample initially increases and subsequently drops at each age. With a carbide slag level of 8%, the combination of CaO in the slag and water glass stimulated the slag and micron silica, leading to the formation of gel substances such C-S-H and C-A-S-H. The soil particles exhibited increased density as a result of the cohesive properties of the gel products. Following a maintenance period of 28 days, the sample’s compressive strength rose to 2280 kPa. When the carbide slag level exceeds 8%, the presence of Ca(OH)_2_ in the mixture leads to the formation of carbonates, such as calcite, during the carbonization process. The organic matter subsequently undergoes a reaction with the Ca(OH)_2_ produced during the hydration of the mixture, leading to the formation of a highly soluble complex. As a result, only a limited quantity of calcium ions in the pore solution participate in the pozzolanic reaction, hence reducing the formation of gel reaction products such C-S-H.

## 1. Introduction

Large quantities of dredged sludge (DS), characterized by low strength, high water content, and poor bearing capacity would be generated due to port development, river dredging, and water environment treatment projects [1, 2]. The mud disposal method employed in the dredging project not only destroys the ecological environment but also occupies a large amount of land. To address these DS, researchers have enhanced sludge treatment by employing curatives and stimulants, such as industrial waste, to achieve waste treatment through the use of waste [3–6]. Utilizing different curing agents and alkaline activators to cause the solidification of sludge is a highly successful method to improve the strength of solidified DS [7, 8]. Industrial solid waste such as steel slag, slag, carbide slag and fly ash, can serve as a curing agent for DS. This alternative can partially substitute the conventional cement curing agent, which is associated with high pollution, high emission and high energy consumption. DS solidification treatment has emerged as a hotspot of research [9–12].

Organic materials are found in the DS formed as a result of river dredging. Fulvic acid, the main component of organic materials, has a negative impact on the microstructure and strength properties of DS [13–16]. Fulvic acid is an organic polymer that occurs naturally and has a mildly acidic nature [17]. The strong water absorption capability of organic soil is directly influenced by its internal structure, resulting in a high moisture content retention capacity, low strength, and poor permeability. Experimental research has demonstrated that the presence of fulvic acid on the soil particle surface slows down or even prevents the bonding between soil particles and gel material, leading to a greater negative impact on soil healing [18, 19]. Du et al. [20] used sodium humate to prepare artificial organic soil. As the amount of organic matter in the sample rose, the moisture content after curing likewise increased, resulting in a considerable loss in sample strength. Yunus et al. [21] investigated the effect of humic acid on the stability of lime-stabilized organic clay. When the concentration of humic acid exceeded 1.5%, the strength of lime-stabilized organic clay experienced a more pronounced decline. Cement is commonly used to solidify waste sludge, and it is one of the widely used cementitious materials. The presence of humic acid in organic-rich silt affects the solubility of cement particles. This is because humic acid, being a weak acid, neutralizes the alkaline environment necessary for the hydration reaction. As a result, the formation of gel products like hydrated calcium silicate (C-S-H) and hydrated calcium aluminate (C-A-H) is reduced [22–24]. In their study, N. Ghasabkolaei et al. [25, 26] investigated the use of lime as an alkaline exciter and cement as a curing agent to improve the strength of cement-cured dredge sludge (CDS). Their findings revealed that the use of lime can promote the synthesis of gel substances. Lime, being an alkaline activator, can counteract the effects of humic acid in waste sludge and create a highly alkaline environment that promotes the hydration reaction in the mixture. Nevertheless, when used as a foundation material with soft soil, it will adversely affect the security of the nearby groundwater [27, 28]. The widespread utilization of lime and cement for the purpose of solidifying DS will result in the depletion of non-renewable energy resources and give rise to significant air pollution issues [29–31]. It has been reported that the comprehensive utilization rate of industrial waste is below 55% [32–34], which leads to a series of problems such as high solid waste cost, waste of resources and environmental pollution. Cristelo et al. [35] employed a combination of lime and slag for the treatment of marine clay, resulting in a long-term compressive strength superior to that of cement solidified clay. According to Wang et al. [36, 37], the unconfined compressive strength (UCS) of silt soil solidified with 6% slag-carbide slag-steel slag is equivalent to that of cement soil at the same age. The presence of silica in water glass has a delaying impact on the reaction, which promotes the development of more compact hydration products and enhances the strength at a later stage [38]. Hu et al. [39] examined the effect of slag on the cracking resistance of concrete using an annular restraining device. Their findings revealed that the addition of slag to the concrete increased its drying shrinkage and reduced its resistance to breaking. The research and production of low-carbon and environmentally friendly materials has gained significant interest in recent years. The utilization of industrial waste in the treatment of DS has also garnered broad attention. The effects of adding organic matter, cement, and carbide slag to improve the strength and microstructure of cured silt with a high organic matter content have not been thoroughly studied. This study focuses on employing a composite exciter consisting of slag, micron silica, water glass, and carbide slag. Table 1 summarizes the existing research work.

**Table 1:** Summary of selected research work.

Authors	Research work
Du [20]、Yunus [21]	Effect of organic matter content on sludge.
N. Ghasabkolaei [25]	The cement and lime solidified sludge’s strength changing law is examined. Environmental pollution is not taken into account in the study process.
Cristelo [35]	The mixture of lime and slag has an effect on the strength of marine clay, but lime has a greater pollution to the environment.
Wang [36]、Wang [37]、Hu [39]	The detrimental effects of organic content on solidified silt soil were disregarded in favor of examining the impact of industrial wastes like slag and carbide slag on the soil’s strength.
Gong (the present work)	Using slag, micron silicon, water glass, and carbide slag as composite activators, the effects of cement, organic matter, and carbide slag content on the strength and microstructure of solidified high organic matter sludge were investigated in order to create a high-efficiency curing agent that is low-carbon, energy-efficient, and environmentally benign.

Based on this, alkaline exciters such as carbide slag and water glass were selected, and slag and micronized silica were utilized as precursors to create an eco-friendly and low-carbon composite curing agent. This paper used granulated blast furnace slag powder as the primary raw material to enhance the curing process of waste high organic matter silt, with the assistance of alkaline calcium carbide slag micronized powder and water glass. An analysis was conducted on the properties and mechanism of solidified sludge using macro and micro test methods. The internal micro process underlying the development of a dense microstructure and the enhancement of mechanical characteristics following the solidification of organic sludge was uncovered by this investigation. These results provide a foundation for improving the viability and efficiency of solidification of organic sludge. The slag and carbide slag used in this study are economical, environmentally benign, and low-carbon materials that have the potential to achieve a high rate of solid waste utilization. This improves the use of slag and carbide slag and lessens dependency on expensive activators.

## 2. Materials and methods

### 2.1. Materials

#### 2.1.1. Sludge

The test sludge was collected at a river channel dredging site in Xuzhou City. It was carefully packed with plastic wrap after sampling and transported to the laboratory for further testing. Following the process of drying, crushing, and screening, the experimental raw materials were acquired for future use. The physical parameters of the material were examined following the guidelines outlined in the GB/T50123-2019 Standard for Geotechnical Test Methods [40]. The findings are presented in Table 2.

**Table 2:** Basic physical property indicators for sludge.

Parameters	liquid limit (%)	plastic limit (%)	liquidity index (I_L_)	plasticity index (I_P_)	water content (%)	PH value	organic content (%)	natural density (g·cm^-3^)
Value	47.6	26.6	2.05	23.2	70	7.16	2.15	1.85

**Fig 1:**
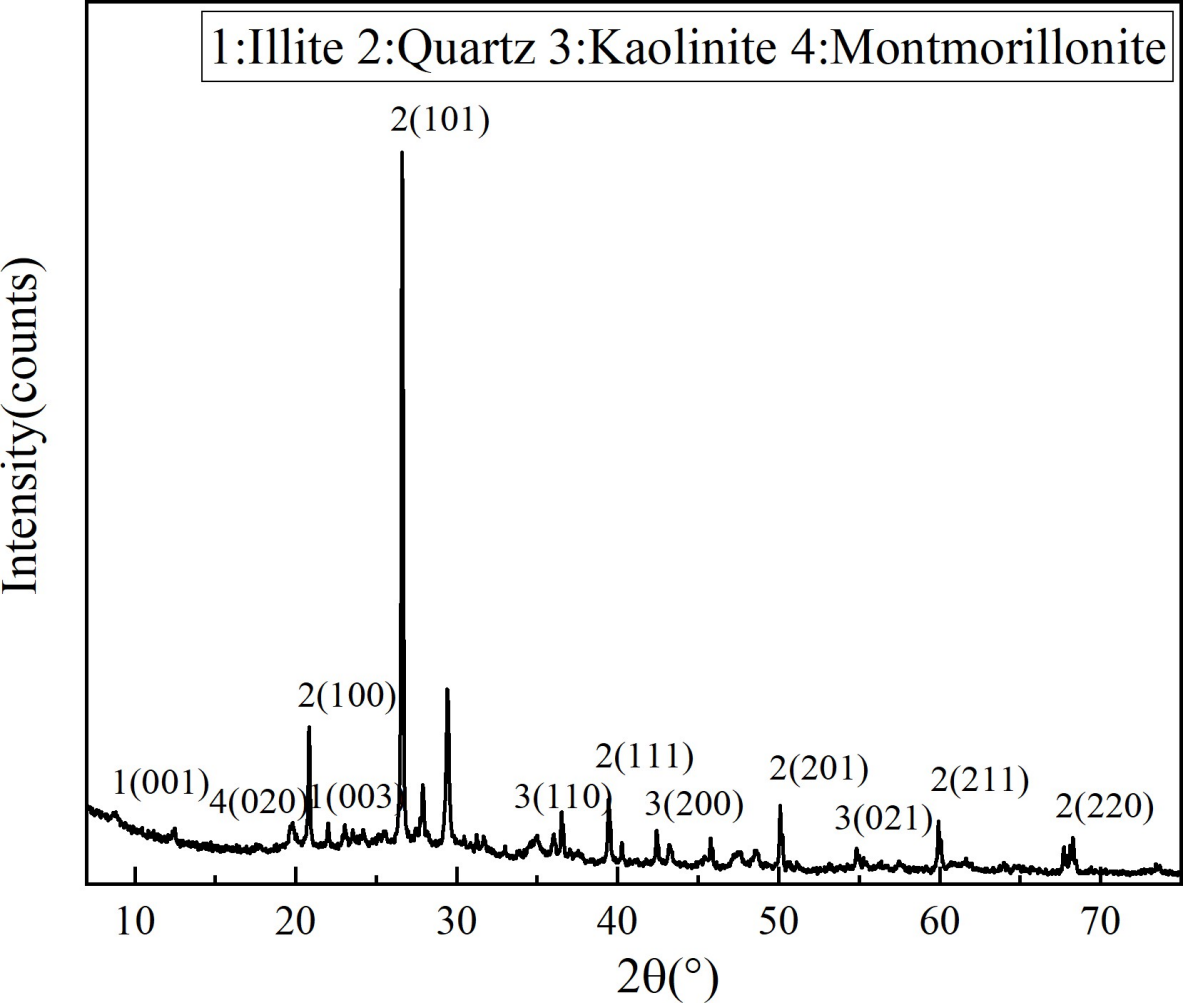
XRD pattern of sludge sample.

The XRF (X-Ray Fluorescence) and XRD test results of the silt sample can be found in Table 3 and Fig 1, respectively. Table 3 reveals that the silt’s total mass is comprised of four primary chemical components: SiO_2_, Al_2_O_3_, Fe_2_O_3_, and CaO. These components collectively account for 88.49% of the silt’s total mass. The XRD patterns of the silt sample reveal that quartz, kaolinite, montmorillonite, and illite are the predominant mineral compositions of the silt.

**Table 3:** Chemical composition of sludge samples (%).

chemical composition	SiO_2_	Al_2_O_3_	Fe_2_O_3_	CaO	MgO	K_2_O	Na_2_O	SO_3_	P_2_O_5_	Others
Value (%)	61.24	16.22	6.01	5.02	2.81	1.07	3.75	0.11	0.29	3.48

#### 2.1.2. Curing agent and activator

The curing agent selected was common Portland cement, while the precursors used were slag and micron silicon. Water glass and carbide slag were employed as alkaline exciters. The experimental cement was procured from a cement plant located in Xuzhou City. The slag and carbide slag were obtained from an iron and steel industry situated in Zhengzhou City. Micron silicon was acquired from a new material technology company. Water glass was purchased from a material factory in Zhengzhou. The slag contains a high concentration of SiO_2_ and CaO, reaching 76.13%. Calcium carbide slag has an even higher CaO content, reaching 81.95%. Water glass is made from solid powder of sodium silicate pentahydrate, which appears as a white powder. It consists of 28.25% SiO_2_ and 29.5% Na_2_O. Cement has a high content of SiO_2_ and CaO, measuring 85.88%. Lastly, micron silicon contains an extremely high concentration of activated SiO_2_, reaching 99.95%. Table 4 displays the chemical constituents of the raw materials that were tested.

**Table 4:** Chemical composition of experimental materials.

Raw materials	Percentage content of the sample (%)							
	SiO_2_	Al_2_O_3_	CaO	Fe_2_O_3_	MgO	Na_2_O	SO_3_	K_2_O
Slag	35.78	11.86	40.35	0.83	5.55	0.35	-	-
Carbide slag	10.11	0.48	81.95	2.02	2.31	-	-	-
Water glass	28.25	-	-	-	-	29.50	-	-
Cement	21.77	4.09	64.11	3.33	1.41	0.06	2.66	0.62
Micron Silicon	99.95	-	-	-	-	-	-	-

### 2.2. Mix design and sample preparation

Based on the previous study [41], the experimental proportioning design is displayed in Table 5. Three sets of experimental programs were set up: Experiment Series I to examine the effect of organic matter content as well as cement dosing on cement-cured silt; Experiment Series II to investigate the effect of carbide slag dosing on silt sample; and Experiment Series III to explore the effect of organic matter content on silt sample when composite excitation was performed. The experiment Series I consisted of six distinct experimental groups. the organic matter doping levels were set at 0%, 1%, 2% and 3%, while the cement doping level were set at 20%, 25% and 30%, respectively. Experimental series II consisted of five distinct experimental groups, each receiving a different dosage of carbide slag: 4%, 6%, 8%, 10%, and 12%. Experimental series III consisted of four distinct experimental groups, each receiving varying dosages of organic matter (0%, 1%, 2%, and 3%), a 3% dosage of water glass, and an 8% dosage of carbide slag. The quantities of cement, slag, water glass, carbide slag, organic matter, and water in Table 5 are determined by the mass ratio of dry soil. The code P25J2 in the table represents a cement dosage of 25% and an organic matter dosage of 2%. The other sample numbers have a similar meaning.

**Table 5:** Mix design.

Series	Cement (%)	Slag /SiO_2_	Water glass (%)	Carbide slag (%)	Organic matter (%)	C_W_ (%)	C_D_ (days)	Number
Ⅰ	25				0	70	7、14、28	P25J0
	25				1			P25J1
	25				2			P25J2
	25				3			P25J3
	20				2			P20J2
	30				2			P30J2
Ⅱ		15/5	3	8	0	70	7、14、28	SJ0
		15/5	3	8	1			SJ1
		15/5	3	8	2			SJ2
		15/5	3	8	3			SJ3
Ⅲ		15/5	3	4	2	70	7、14、28	KD4
		15/5	3	6	2			KD6
		15/5	3	8	2			KD8
		15/5	3	10	2			KD10
		15/5	3	12	2			KD12

Note: C_W_: water content; C_D_: curing time

**Fig 2:**
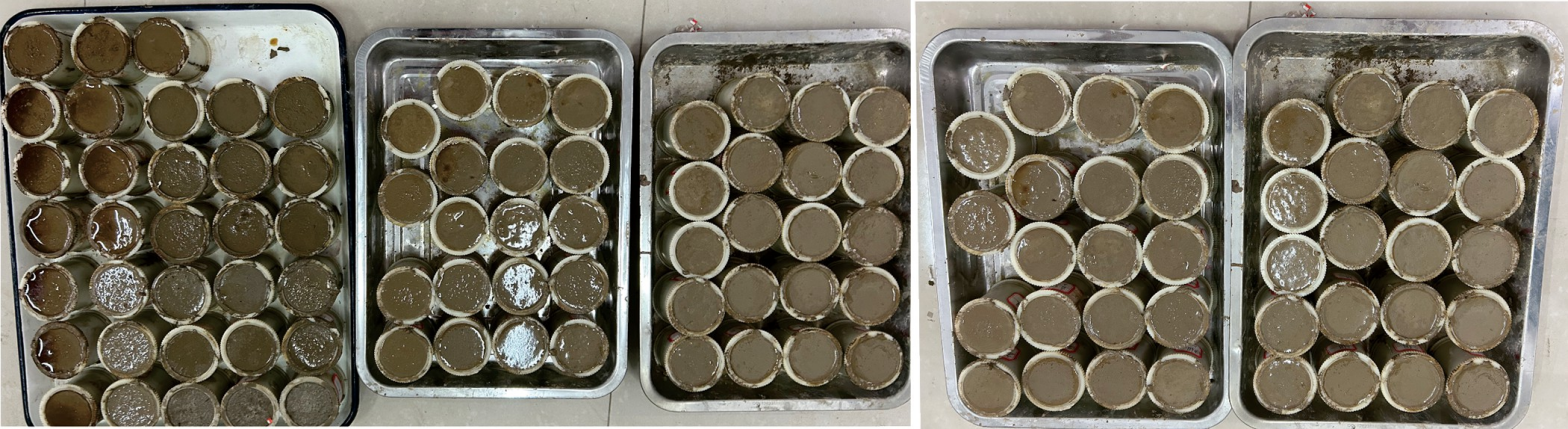
Solidified sludge sample.

The silt that was present on the site was dried at 60°C and then crushed in order to remove any pollutants. Different curing agents and exciters were experimentally configured and mixed with the dry soil, which has a high concentration of organic matter, to create a soil sample with a water content of 70%. The silt mixture was poured into PVC cylindrical molds in three stages, with each layer being filled after 1 minute of oscillation. Following the processing of the sample, a layer of plastic wrap was applied to the surface of the mold in order to inhibit the evaporation of water. The samples were placed in a standardized curing chamber at a controlled temperature of 20°C ± 1°C. After then, the samples were taken out of the molds and allowed to cure for three specific time periods: 7 days, 14 days, and 28 days. Fig 2 displays the sludge samples.

### 2.3. Experimental method

The test was conducted using a strain-controlled UCS testing machine. A loading rate of 1 mm/min was used. The test was performed at 7, 14, and 28 days of age. The test results were obtained by averaging the values of three parallel samples. XRD was used to test the mineral composition of the dry powder of the sample, the scanning range was 2*θ* = 5-80°, and 5°/min was the scanning rate. The micro morphology of the dried samples’ natural slice was examined using SEM. TG analysis tested the samples for hydration products.

## 3. Results and analysis

### 3.1. Effect of organic matter content on CDS strength

**Fig 3:**
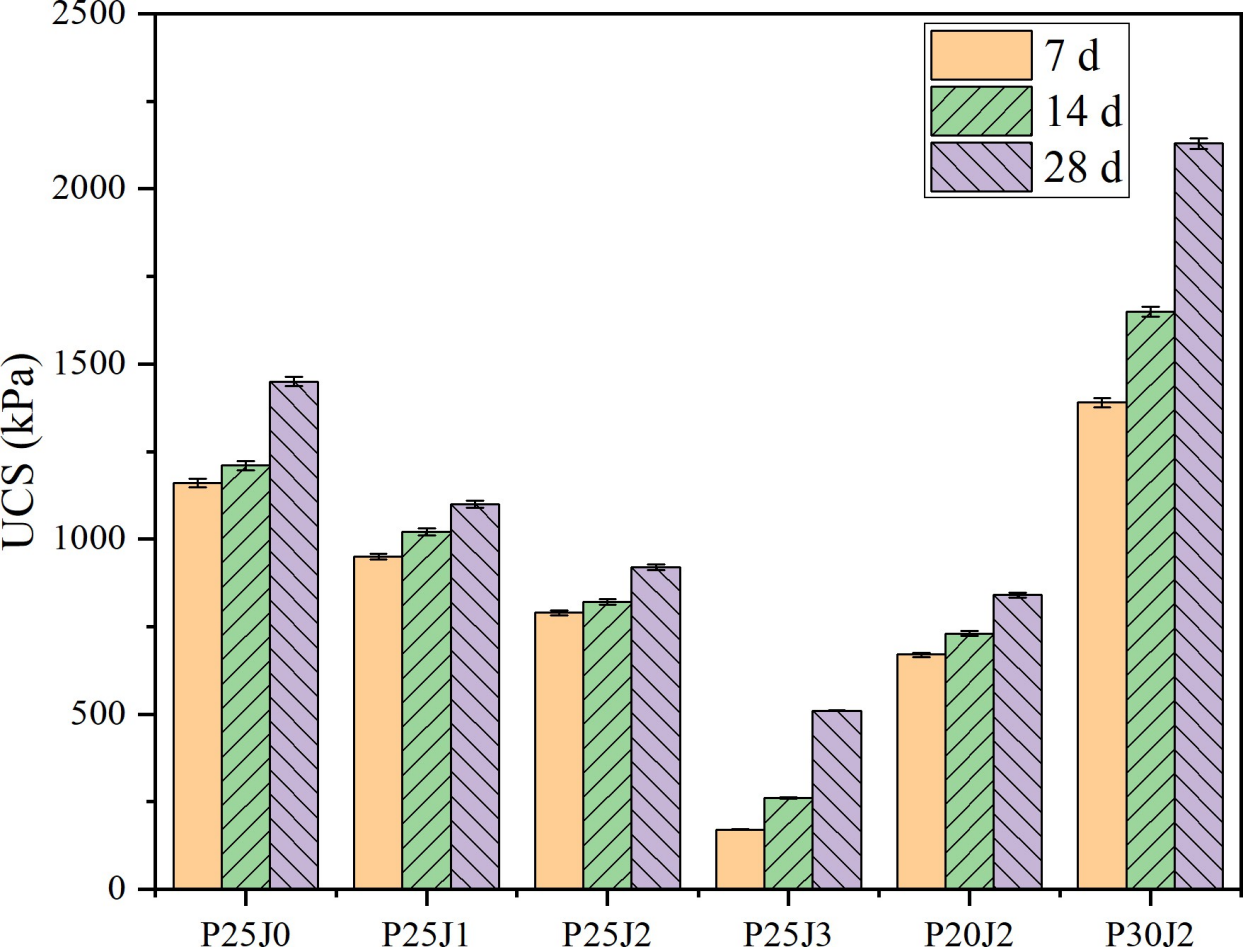
Effect of organic matter and cement content on CDS strength. The error bar in the figure represents the standard deviation of multiple averages (data is reflected in the S1 Table of the S1 File).

Experiment series I explored the effects of organic matter and cement content on cement-cured silt. The experimental results are shown in Fig 3. At a cement curing agent concentration of 25%, the strength of the silt sample exhibited a notable decline as the proportion of organic matter increased. After curing for 7 days, the silt samples containing 0%, 1%, 2%, and 3% organic matter had unconfined compressive strengths of 1160, 950, 790, and 170 kPa, respectively. The early strength of the silt samples exhibited a clear trend of decreasing as the proportion of organic matter increased. After being subjected to a maintenance period of 28 days, the unconfined compressive strength of the sample was measured to be 1450, 1100, 920, and 510 kPa. The strength of the sample exhibited the same pattern of change as mentioned earlier. When the organic matter dosage was controlled at 2%, the strength of the silt sample increased as the dosage increased. At the 7th day, the strength was 670 kPa for a 20% dosage, 790 kPa for a 25% dosage, and 1390 kPa for a 30% dosage. At the 14th day, the strength was 730 kPa, 820 kPa, and 1650 kPa for the respective dosages. Finally, at the 28th day, the strength was 840 kPa, 920 kPa, and 2130 kPa for the respective dosages. The study shown that augmenting the proportion of cement can effectively counteract the adverse impact of organic materials.

### 3.2. Effect of organic matter content on DS strength during composite excitation

**Fig 4:**
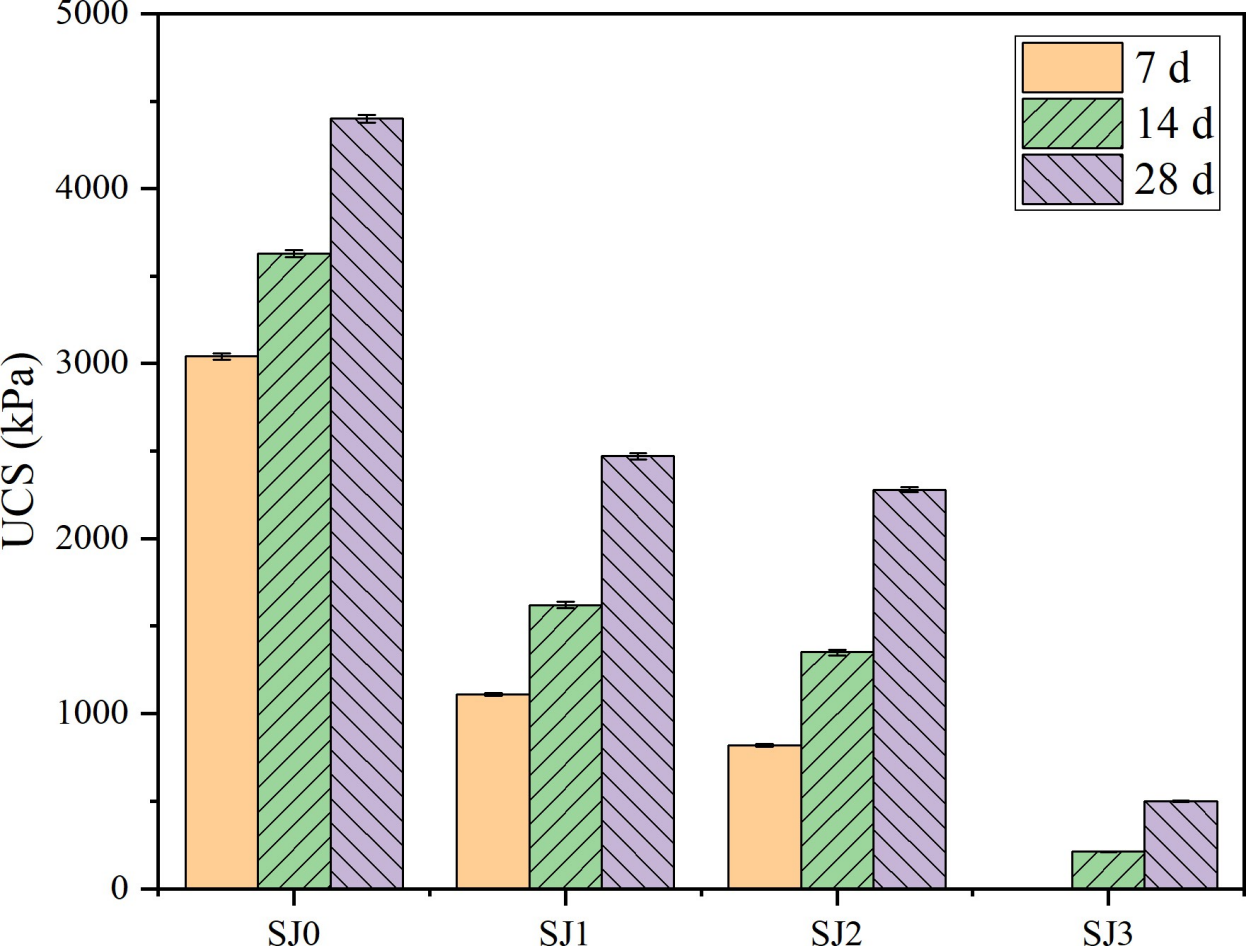
Effect of organic matter content on DS strength during composite excitation. The UCS is obtained from the average of multiple parallel specimens. (data is reflected in the S2 Table of the S1 File).

Experiment series II investigated the effect of organic matter content on DS strength during composite excitation. The experimental results, depicted in Fig 4, indicate that the organic matter content increased from 0% to 3%. Additionally, the strength of the sample at each age exhibited a declining pattern. At an organic matter level of 3%, the sample was unable to be removed from the mold after a curing period of 7 days. After 28 days of maintenance, the strength of SJ0 was 8.8 times greater than that of SJ3. Evidence indicates that soil organic matter can significantly diminish the engineering characteristics of silt soil and impact its chemical curing efficacy.

### 3.3. Effect of carbide slag dosing on DS strength during composite excitation

**Fig 5:**
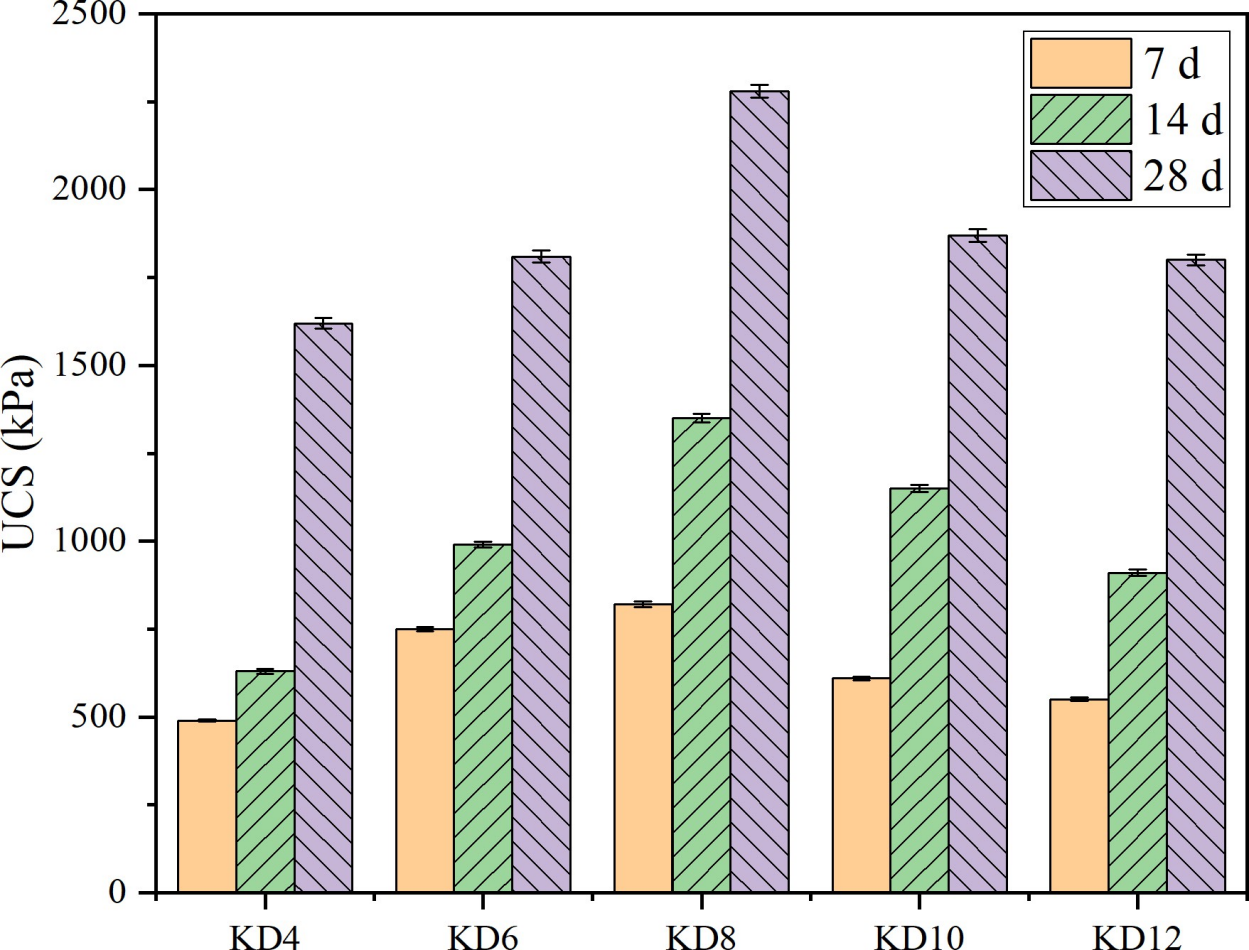
Effect of carbide slag dosing on DS strength during composite excitation. (data is reflected in the S3 Table of the S1 File).

Experiment series III explored the effect of calcium carbide slag dosing on the strength of DS during composite excitation. Fig 5 displays the outcomes of the experiment. The strength of the sample at each age exhibited a progressive increase from 4% to 12%, followed by a subsequent decline. The highest strength of the high organic matter silt sample, which contained 2% of the precursor, was observed when the precursor dosage was 20%, water glass dosage was 3%, and the dosage of calcium carbide slag was 8%. After 14 days of conditioning, the strength of sample KD8 was 1.65 times more than the strength of sample P25J2. This further demonstrates the superior performance of the composite exciter in overcoming the impact of high organic matter silt. It can be seen that the new composite curing agent can partially replace the cement curing agent with high pollution and high energy consumption.

### 3.4. Micro-mechanism analysis

Following the UCS tests, the specimens’ microstructure and micromorphology were examined. The following specimens are mostly analyzed using XRD, SEM-EDS, and TG.

#### 3.4.1. XRD analysis

**Fig 6:**
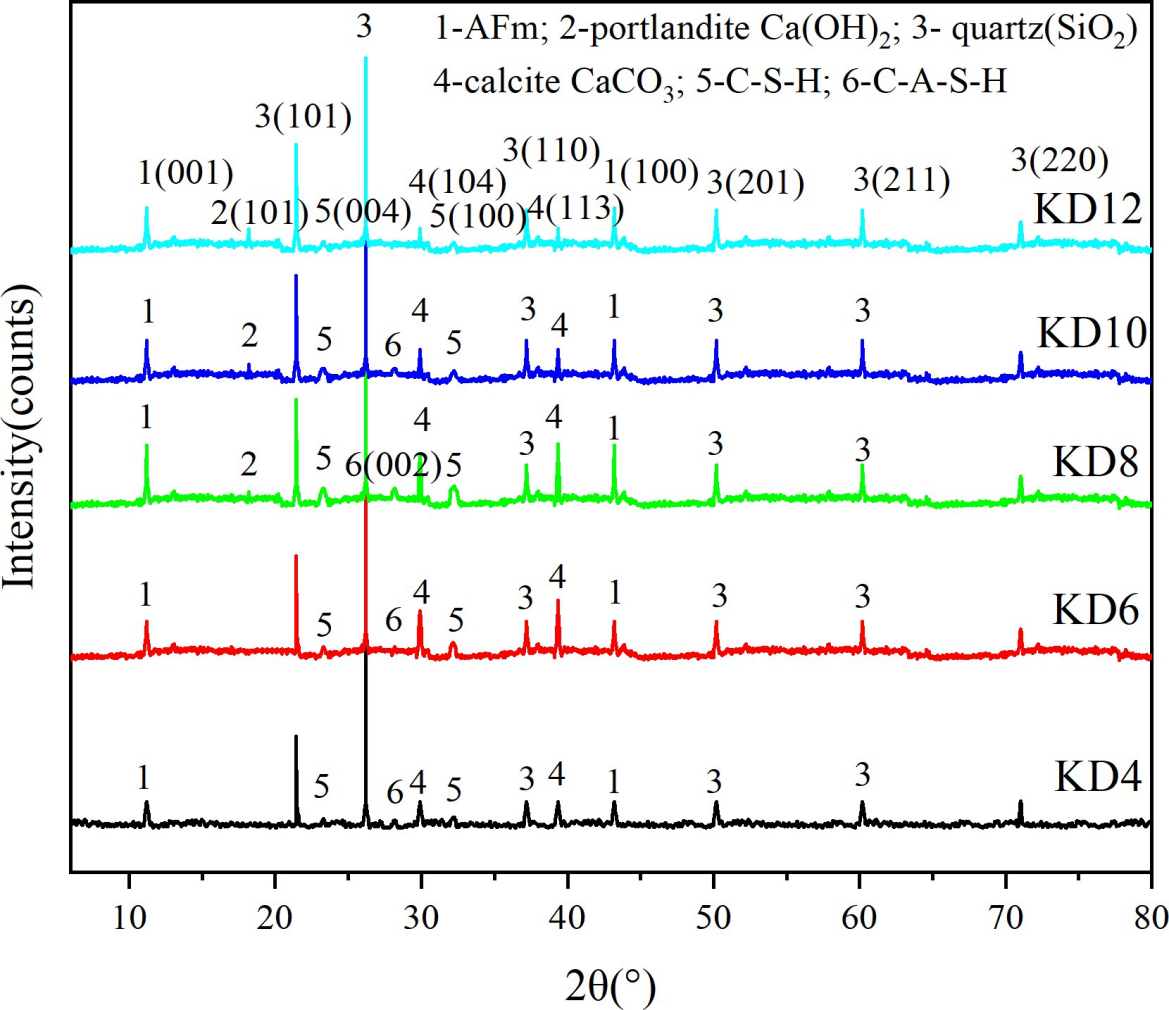
The XRD spectra of cured sludge with different dosages of carbide slag for 28 days. XRD measures the mineral composition of the specimens. (data is reflected in the S4 Table of the S1 File).

An analysis was conducted on the mineral composition of the KD sample that underwent rapid carbonization for a duration of 28 days. The principal mineral crystal phases were identified and marked, and the XRD spectra obtained are displayed in Fig 6. The KD sample exhibited distinct diffraction peaks at 2θ angles of 11° and 43°. These peaks were carefully analyzed and found to be in accordance with the characteristic diffraction peaks of the monosulfur type hydrated calcium sulfoaluminate (AFm). This suggests the presence of AFm in the hydration products, which is one of the factors contributing to the increased strength of hardened bodies [42]. Slag and micro silica contain a large amount of reactive SiO_2_. Calcium carbide slag and water glass generate a strong alkaline environment. The abundant presence of OH^-^ and Ca^2+^ in the aqueous state facilitates the hydration reaction of slag. The XRD spectrum shows the presence of a convex peak near 30°, which indicates the formation of an amorphous C-A-S-H gel [43] in a very alkaline environment. This peak can be attributed to the distinctive peaks of the C-A-S-H or C-S-H amorphous gel structure. When the calcium carbide slag dosage was below 8%, no Ca(OH)_2_ crystals were detected in the carbonation products of the sample. However, carbonate crystals such as calcite were observed. When It is worth noting that the weight loss peak at 698°C in the TG analysis correlated to the presence of calcite. This finding confirms that the Ca(OH)_2_ in the carbonation zone was depleted and additional carbonate crystals, such as CaCO_3_, were formed to facilitate the development of UCS after 28 days of rapid carbonation. Preliminary evidence suggests that calcium carbide slag and water glass create a favorable and potent alkaline environment for slag and micronized silica. Consequently, they serve as effective alkaline stimulants during the hydration phase of the system. When the amount of calcium carbide slag reaches or exceeds 8%, Ca(OH)_2_ experiences depletion followed by crystallization and generation. Simultaneously, the dosage of calcium carbide slag speeds up the carbonation reaction. However, an excess of quicklime causes the slurry to expand and increases porosity, which obstructs the growth of UCS. Table 6 shows the structural parameters of the samples.

**Table 6:** The structural parameters of the samples were calculated according to the XRD results.

Description of sample	The hkl index	Lattice constant (A)	Crystallite size (nm)	Strain (×10^−3^)
Monosulfur type hydrated calcium sulfoaluminate (AFm)	Crystal face (001)	11.8	27.7	0.67
Calcium hydroxide (Ca(OH)_2_)	Crystal face (001)	4.9	33.4	0.58
SiO_2_ (Quartz)	Crystal face (100)	4.26	40.7	0.49
CaCO_3_ (Calcite)	Crystal face (104)	6.37	39.4	0.34
C-S-H (Mullite)	Crystal face (002)	22.0	23.4	0.78
C-A-S-H	Crystal face (001)	12.7	26.3	0.64

#### 3.4.2. SEM-EDS analysis

**Fig 7:**
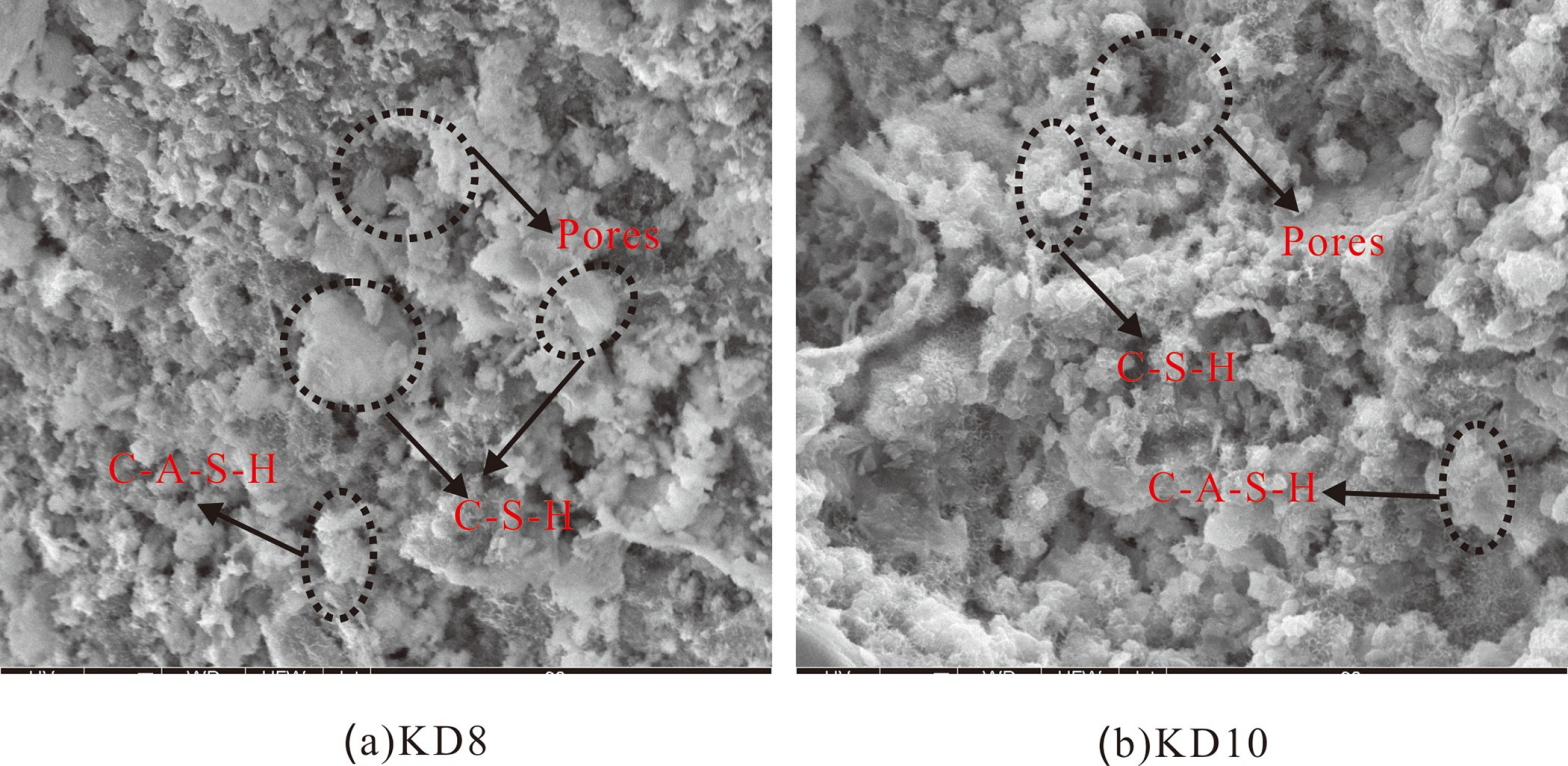
SEM photographs of sample KD8 and KD10. The micro-morphology structure of KD8 and KD10.

**Fig 8:**
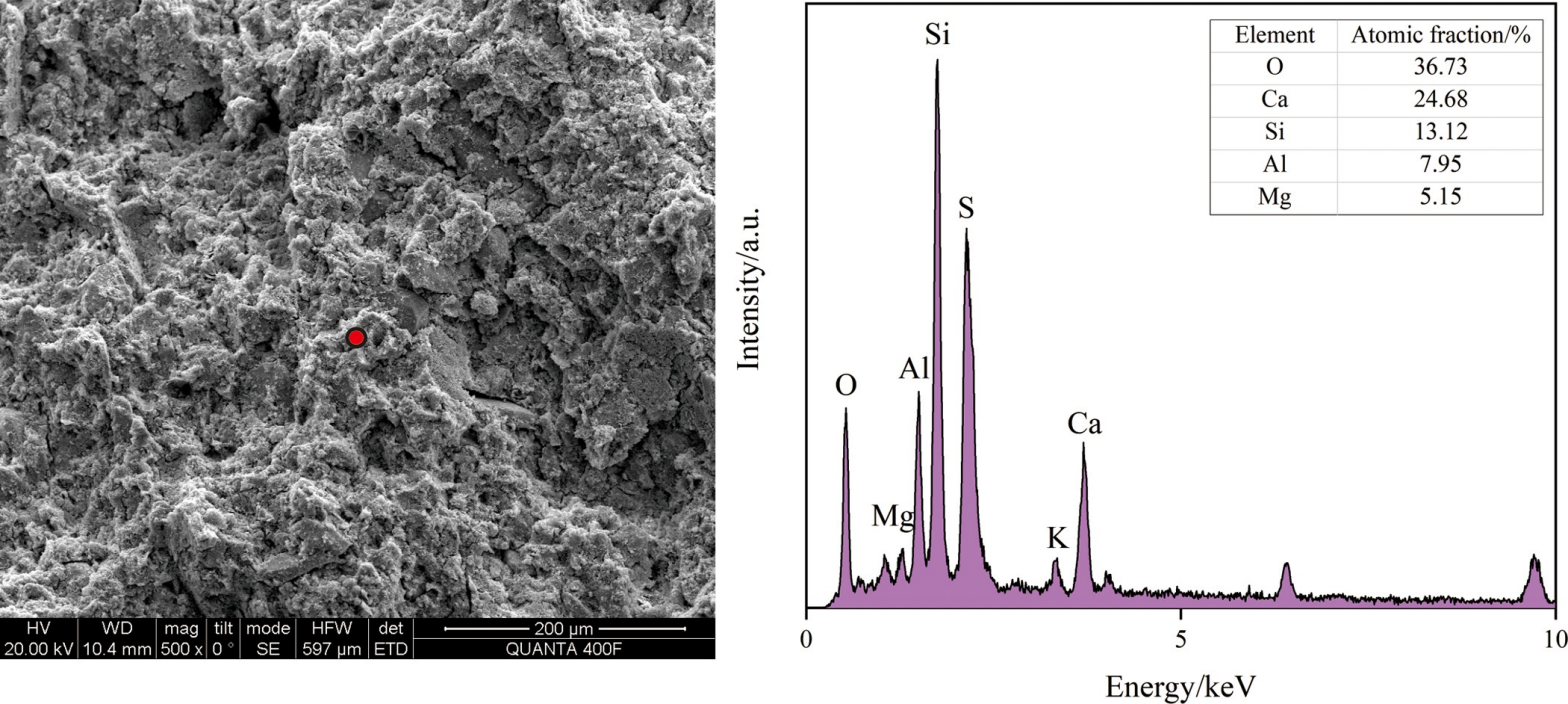
SEM photo EDS analysis of sample KD8. Elemental composition of KD8. (data is reflected in the S5 Table of the S1 File).

**Fig 9:**
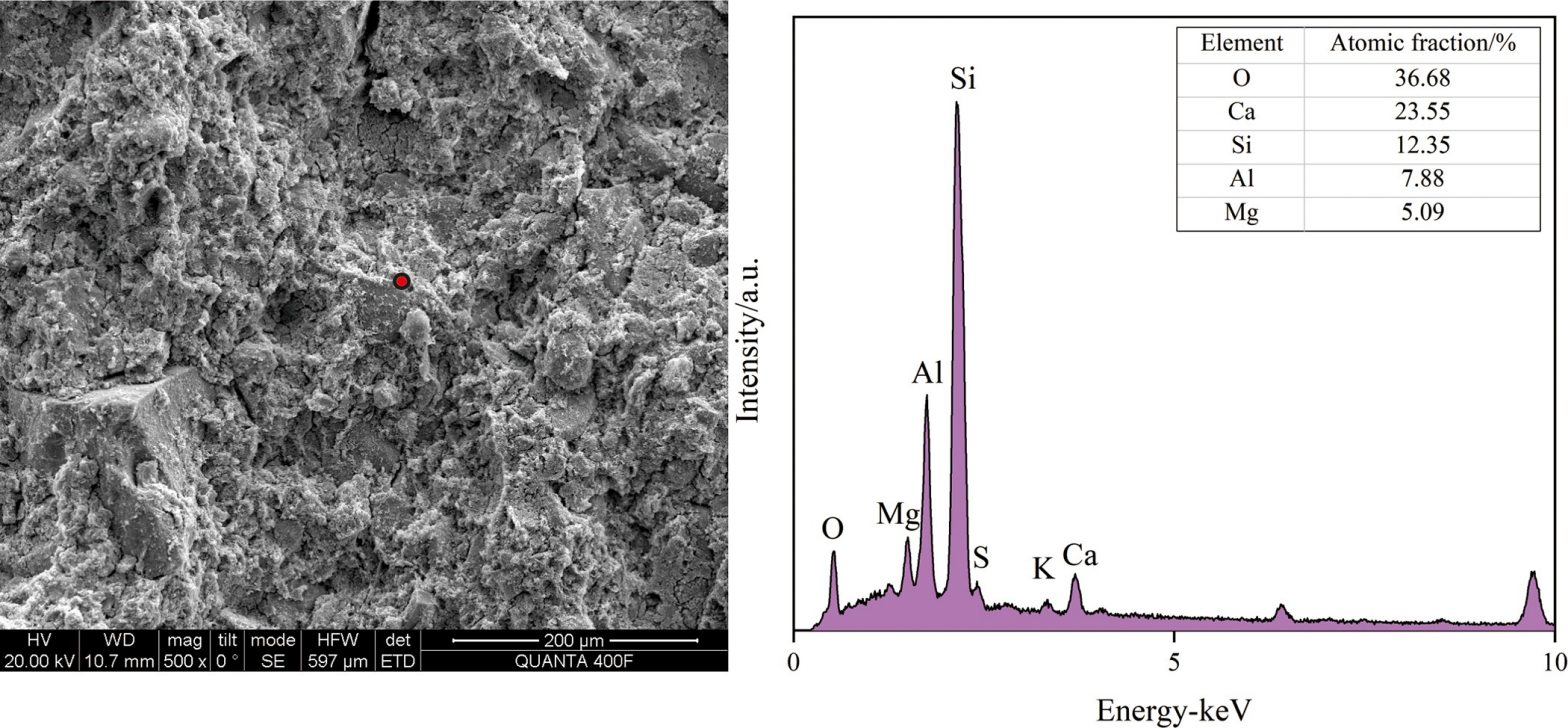
SEM photo EDS analysis of sample KD10. Elemental composition of KD10. (data is reflected in the S6 Table of the S1 File).

Fig 7 shows the SEM photos of representative sample KD8 and sample KD10. SEM analysis of the flaky particles in the crushed sample reveals a significant presence of colloids throughout the matrix structure, with hydrated particles also being disseminated around the colloids. The gel discovered in the hit points has the main constituent elements of O, Mg, Si, Al, and Ca, as determined by EDS analysis. The elemental ratios observed are consistent with the features of the C-A-S-H and C-S-H gels [44, 45]. As shown in Figs 8 and 9, the hydration products of both KD8 and KD10 contain C-A-S-H and C-S-H gels, which may be identified by the prominent “bun peaks” observed in the XRD patterns of the samples. The presence of a “bun peak” in the XRD pattern of the sample confirms its convex packaging. During the analysis of the chemical composition of slag and calcium carbide slag raw materials, it was found that both contain a certain amount of SO_3_ and Al_2_O_3_. However, calcium carbide slag has a higher concentration of Ca. When the hydration process occurs, a portion of SO_4_^2-^ and a large quantity of Ca^2+^ are produced. In an alkaline environment, AFm crystals and Ca(OH)_2_ crystals are formed. By combining this information with XRD analysis, it can be inferred that the hydration products consist of hexagonal lamellar crystals, primarily Ca(OH)_2_, with a small amount of AFm. The study discovered that AFm crystals significantly contribute to the improvement of the sample’s curing strength [46].

#### 3.4.3. Thermogravimetric analysis

**Fig 10:**
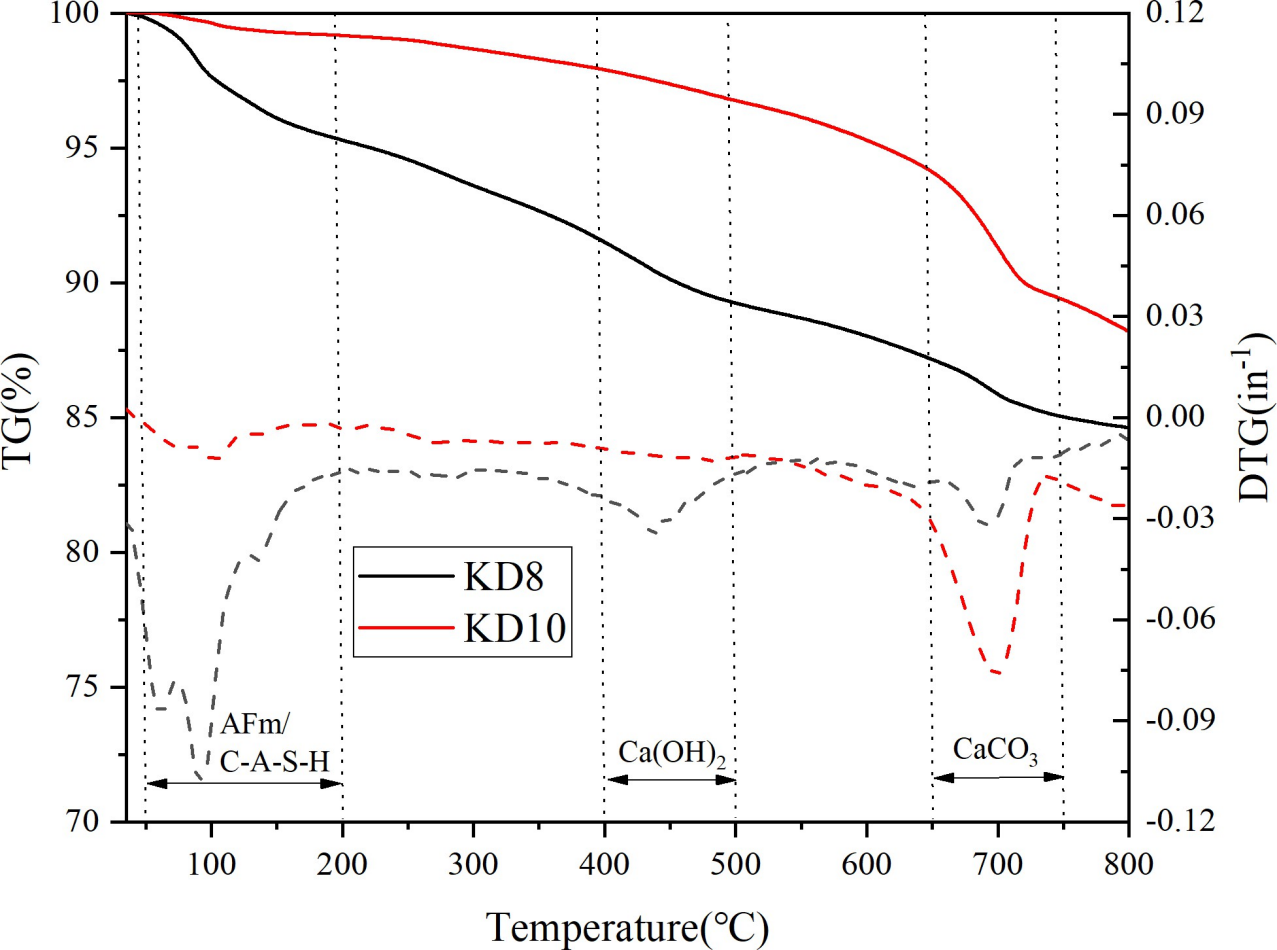
TG-DTG curves of sample KD8 and KD10. TG reflects the mass loss of the sample caused by the change of temperature. (data is reflected in the S7 Table of the S1 File).

Thermogravimetric analysis was conducted to gain a deeper understanding of the composition and mechanism of action of the hydration products in the silt curing body. The thermogravimetric curves of representative sample KD8 and KD10 were expressed as TG. The TG-DTG of sample KD8 and KD10 cured for 28 days is shown in Fig 10. The thermal decomposition of the hydration products represents the mass loss of the sample. Sample KD8 and KD10 were analyzed thermogravimetrically after 28 days of curing by taking 10 mg of each. The thermogravimetric differential curves are denoted by DTG. There were three exothermic peaks seen between the temperatures of 50 and 800°C. The substances responsible for the first exothermic peak were C-S-H and AFm (50–200°C). The mass loss observed in this peak was primarily due to the evaporation of free water and the removal of bound water from the AFm, C-S-H, and C-A-H gels [47]. The second exothermic peak, occurring at 200–500°C, was attributed to the decomposition of Ca(OH)_2_ [48]. This suggests that Ca(OH)_2_ is still being generated or present during the hydration process. The substance responsible for the third exothermic peak, observed at 500–750°C, is CaCO_3_. This peak is typically associated with the thermal decomposition of carbonates [49–51]. Within the temperature range of 50 to 800°C, sample KD8 saw a total weight loss of 14.99% after 28 days of conditioning, while sample KD10 had a total weight loss of 12.12% under the same conditions. This further proves that sample KD8 has more hydration products. Based on the macroscopic analysis, a higher overall weight loss rate indicates a greater presence of hydration products, therefore confirming that the dosage of calcium carbide slag has a significant impact on the formation of hydration products. This aligns with the findings of the prior strength study.

## 4. Conclusions

The study investigated the impact of organic matter content and calcium carbide slag doping on the strength of DS during composite excitation, based on indoor geotechnical testing, microscopic investigations, and theoretical analysis. The study also examined the changing pattern of DS strength. The key findings are as follows:

(ⅰ) Alkali excitation is improved when carbide slag and water glass are added to slag and micronized silica. When the amount of carbide slag is 8%, the amount of water glass is 3%, and the amount of precursor is 20%, the UCS is larger. The sample’s compressive strength, after 28 days of curing, is 2280 kPa, 150 kPa more than that of the sample that was cured with 30% cement-cured sludge. It demonstrates that the composite excitation agent may partially replace the cement curing agent, which is characterized by high energy consumption and high pollution.

(ⅱ) The primary products of composite excitation, according to the XRD, SEM-EDS, and TG analyses, are C-A-S-H gel, C-S-H gel, and a minor amount of AFm. The main element influencing the sludge cure body’s increased strength is the presence of these hydration products. Meanwhile, when the dosage of carbide slag exceeds 8%, the excess quicklime accelerates the carbonation reaction, and at the same time leads to expansion of the slurry and increase of porosity, which inhibits the formation of UCS.

(ⅲ) During composite excitation, the UCS of the sludge curing body decreases more pronouncedly as the concentration of organic matter rises. The UCS of SJ1 was 4.94 times higher than that of the SJ3 sludge cure body after 28 days of maintenance. The results of the study will provide a strong basis for further research on the sludge remediation with a high organic content.

The two elements of solidification and usage efficiency of high organic matter sludge are the primary focus of the upcoming research work. Enhancing the solidification and usage efficiency of waste sludge and industrial waste residue is the goal of the subsequent research project.

## Supporting information

S1 File Supporting data. (XLS)

S1 Raw image (DOCX)
