# Supplementary material for: Research on the impact of carbide slag content on the strength and microstructure of solidified sludge during composite excitation
Source: PLoS One. 2024 Dec 16;19(12):e0314809. doi: 10.1371/journal.pone.0314809 (PMC11649083; doi:10.1371/journal.pone.0314809)
Supplement: S1 Raw image — (DOCX) [file pone.0314809.s002.docx]

**Fig 1. XRD pattern of sludge sample**


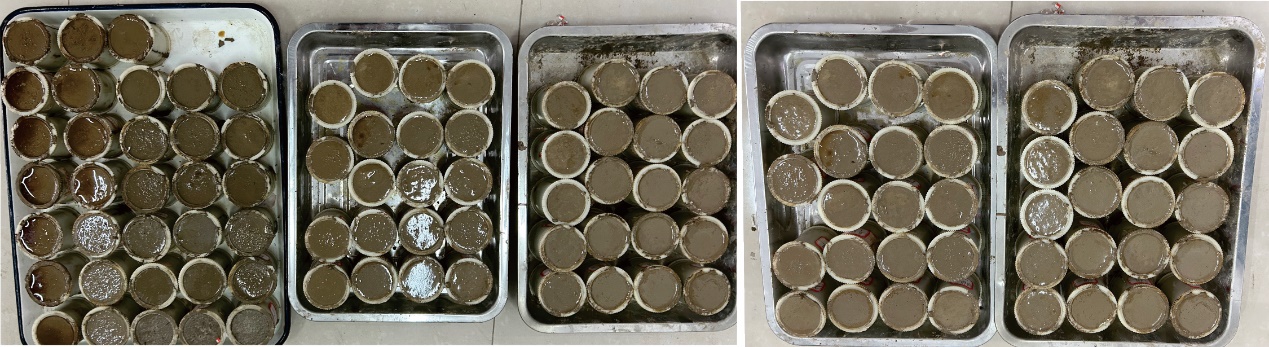


**Fig 2.** **Solidified sludge sample**





**Fig 3. Effect of organic matter and cement content on CDS strength.** The error bar in the figure represents the standard deviation of multiple averages. (data is reflected in the table S1 of the S1 file)





**Fig 4. Effect of organic matter content on DS strength during composite excitation.** The UCS is obtained from the average of multiple parallel specimens. (data is reflected in the table S2 of the S1 file)





Fig 5. Effect of carbide slag dosing on DS strength during composite excitation. (data is reflected in the table S3 of the S1 file)





**Fig 6.** The XRD spectra of cured **sludge** with different dosages of carbide slag for 28 days. XRD measures the mineral composition of the specimens. (data is reflected in the table S4 of the S1 file)


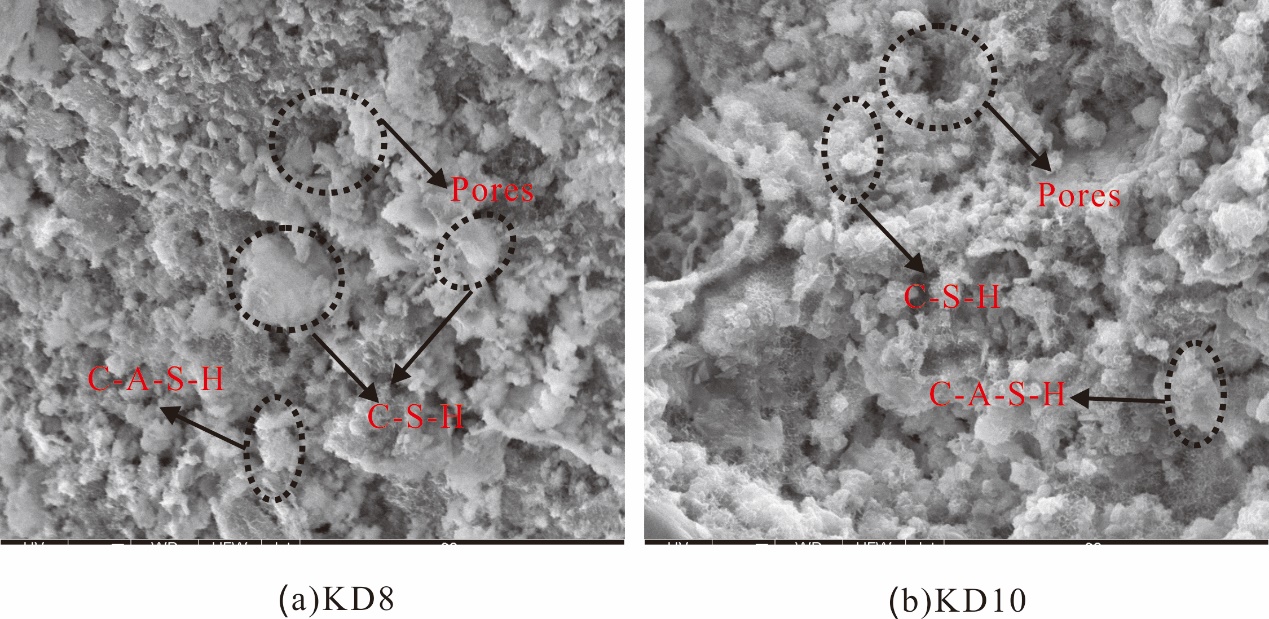


**Fig 7. SEM photographs of sample KD8 and KD10.** The micro-morphology structure of KD8 and KD10.


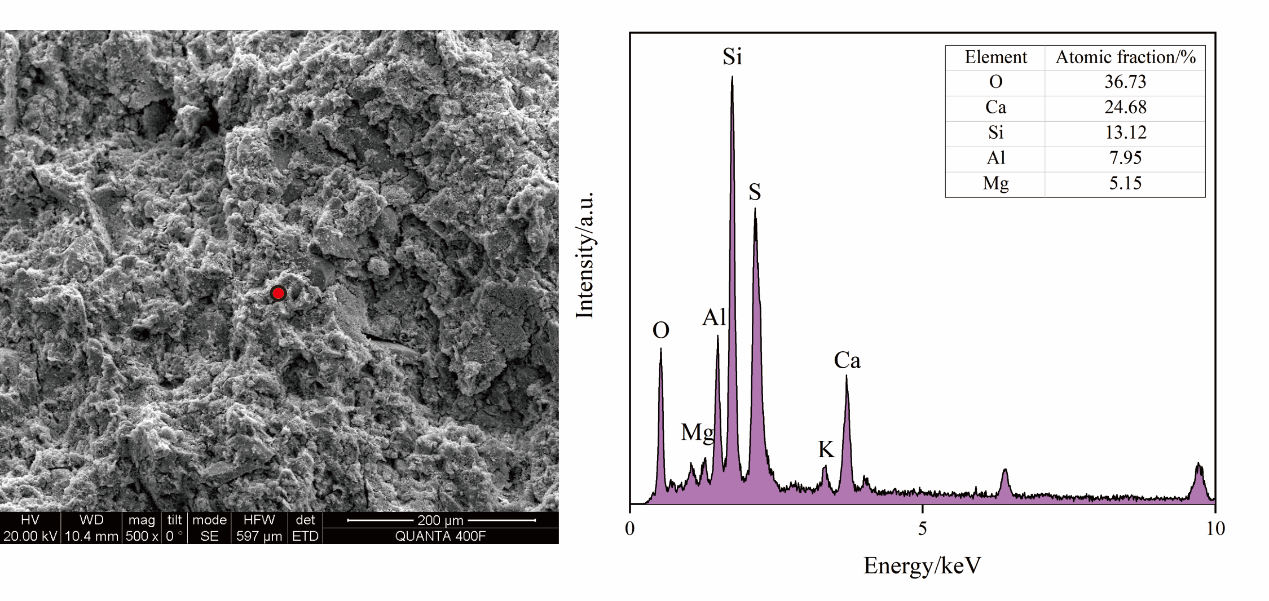


**Fig 8. SEM photo EDS analysis of sample KD8.** Elemental composition of KD8. (data is reflected in the table S5 of the S1 file)


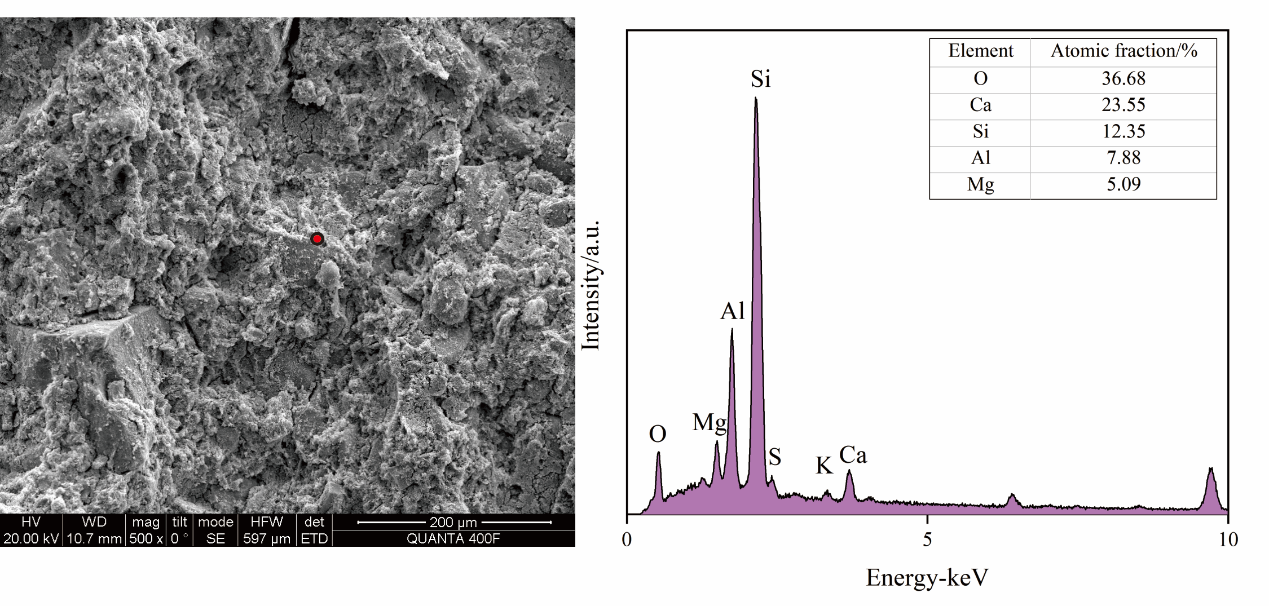


Fig 9. SEM photo EDS analysis of sample KD10. Elemental composition of KD10. (data is reflected in the table S6 of the S1 file)





**Fig 10. TG-DTG curves of sample KD8 and KD10.** TG reflects the mass loss of the sample caused by the change of temperature. (data is reflected in the table S7 of the S1 file)
